# The genetic contribution to the comorbidity of depression and anxiety: a multi-site electronic health records study of almost 178 000 people

**DOI:** 10.1017/S0033291723000983

**Published:** 2023-11

**Authors:** Brandon J Coombes, Isotta Landi, Karmel W Choi, Kritika Singh, Brian Fennessy, Greg D Jenkins, Anthony Batzler, Richard Pendegraft, Nicolas A Nunez, Y Nina Gao, Euijung Ryu, Priya Wickramaratne, Myrna M Weissman, Regeneron Genetics Center, Jyotishman Pathak, J John Mann, Jordan W Smoller, Lea K Davis, Mark Olfson, Alexander W Charney, Joanna M Biernacka

**Affiliations:** 1 Department of Quantitative Health Sciences, Mayo Clinic, Rochester, MN, USA; 2 Department of Psychiatry, Icahn School of Medicine at Mount Sinai, New York, NY, USA; 3 Department of Genetics and Genomic Sciences, Icahn School of Medicine at Mount Sinai, New York, NY, USA; 4 Mount Sinai Clinical Intelligence Center, Icahn School of Medicine at Mount Sinai, New York, NY, USA; 5 Hasso Plattner Institute for Digital Health at Mount Sinai, Icahn School of Medicine at Mount Sinai, New York, NY, USA; 6 Department of Psychiatry, Center for Precision Psychiatry, Massachusetts General Hospital, Boston, MA, USA; 7 Psychiatric & Neurodevelopmental Genetics Unit, Center for Genomic Medicine, Massachusetts General Hospital, Boston, MA, USA; 8 Department of Psychiatry, Harvard Medical School, Boston, MA, USA; 9 Department of Medicine, Division of Genetic Medicine, Vanderbilt University Medical Center, Nashville, TN, USA; 10 Vanderbilt Genetics Institute, Vanderbilt University Medical Center, Nashville, TN, USA; 11 Department of Psychiatry & Psychology, Mayo Clinic, Rochester, MN, USA; 12 Department of Psychiatry, Columbia University and New York State Psychiatric Institute, New York, USA; 13 Department of Population Health Sciences, Weill Cornell Medicine, New York, New York, USA; 14 Clinical and Translational Science Center, Weill Cornell Medicine, New York, New York, USA; 15 Stanley Center for Psychiatric Research, Broad Institute of Harvard and MIT, Cambridge, MA, USA

**Keywords:** Anxiety, depression, electronic health records, polygenic risk

## Abstract

**Background:**

Depression and anxiety are common and highly comorbid, and their comorbidity is associated with poorer outcomes posing clinical and public health concerns. We evaluated the polygenic contribution to comorbid depression and anxiety, and to each in isolation.

**Methods:**

Diagnostic codes were extracted from electronic health records for four biobanks [*N* = 177 865 including 138 632 European (77.9%), 25 612 African (14.4%), and 13 621 Hispanic (7.7%) ancestry participants]. The outcome was a four-level variable representing the depression/anxiety diagnosis group: neither, depression-only, anxiety-only, and comorbid. Multinomial regression was used to test for association of depression and anxiety polygenic risk scores (PRSs) with the outcome while adjusting for principal components of ancestry.

**Results:**

In total, 132 960 patients had neither diagnosis (74.8%), 16 092 depression-only (9.0%), 13 098 anxiety-only (7.4%), and 16 584 comorbid (9.3%). In the European meta-analysis across biobanks, both PRSs were higher in each diagnosis group compared to controls. Notably, depression-PRS (OR 1.20 per s.d. increase in PRS; 95% CI 1.18–1.23) and anxiety-PRS (OR 1.07; 95% CI 1.05–1.09) had the largest effect when the comorbid group was compared with controls. Furthermore, the depression-PRS was significantly higher in the comorbid group than the depression-only group (OR 1.09; 95% CI 1.06–1.12) and the anxiety-only group (OR 1.15; 95% CI 1.11–1.19) and was significantly higher in the depression-only group than the anxiety-only group (OR 1.06; 95% CI 1.02–1.09), showing a genetic risk gradient across the conditions and the comorbidity.

**Conclusions:**

This study suggests that depression and anxiety have partially independent genetic liabilities and the genetic vulnerabilities to depression and anxiety make distinct contributions to comorbid depression and anxiety.

## Introduction

Depression and anxiety disorders are among the most common psychiatric disorders with a lifetime prevalence of 17% and 30%, respectively (Global Burden of Disease Study 2013 Collaborators, 2015). Symptoms of depression and anxiety often co-occur with nearly half of adults with anxiety also reporting depressive symptoms (Kessler et al., 2008). Comorbid depression and anxiety represents a significant clinical and public health concern and is associated with greater severity and earlier age of onset, more severe depression, increased suicidal ideation, poorer antidepressant response, psychosocial impairment, worse course of illness, and increased comorbidity with substance use disorders (Joffe, Bagby, & Levitt, 1993; Nam, Kim, & Roh, 2016; Pollack, 2005; Zhou et al., 2017). Proposed risk factors associated with the comorbidity of depression and anxiety include female sex, younger age, higher educational level, and childhood trauma (de Graaf, Bijl, Smit, Vollebergh, & Spijker, 2002) as well as genetics (Wray et al., 2018).

The etiology of comorbid depression and anxiety disorders remains poorly understood. Epidemiological data suggest that depression and anxiety commonly co-occur because they share underlying liabilities or transdiagnostic factors (Blanco et al., 2013; Kotov et al., 2017; Krueger, 1999). Latent variable techniques applied to the correlational structure of psychiatric comorbidities have consistently identified a broad internalizing factor that includes depression and anxiety disorders (Blanco et al., 2013; Kotov et al., 2017; Krueger, 1999). Clinically, depression and anxiety also have significant overlap with anxiety symptoms being incorporated into some diagnostic criteria for depression (American Psychiatric Association, 2022). Further, dysregulation of the hypothalamic–pituitary–adrenal axis and the role of common neurotransmitters have been implicated in both (Ressler & Nemeroff, 2000; Zorn et al., 2017). These observations, together with shared clinical responses to similar pharmacological (Nutt, 2004) and psychosocial interventions (Craske, 2012) as well as shared genetic risk factors across internalizing disorders, support the common liability hypothesis of depression and anxiety disorders (Blanco, Wall, Feng, & Olfson, 2021).

An alternative possibility is that mood and anxiety have partially or even largely overlapping, but distinct, risk factors and clinical characteristics. Using Research Domain Criteria (RDoC), for example, depression is described as a disorder of impaired reward response, learning, and valuation (Dillon et al., 2014; Greenebaum & Nierenberg, 2020). In contrast, anxiety is described as a dysfunction of threat detection (Dillon et al., 2014; Greenebaum & Nierenberg, 2020). A recent twin study sought to explain both the comorbidity and distinctive nature of the disorders and found that both depression and anxiety have a positive genetic correlation with behavioral inhibition (response to negative stimuli), but only depression has a negative genetic correlation with behavioral activation (response to positive stimuli) (Takahashi, Yamagata, Ritchie, Barker, & Ando, 2021). Risk factors for each disorder also appear to be at least partly distinct such as neuroticism being more strongly linked to depression, arguing against the idea of a fully shared underlying neurobiological liability (Moscati, Flint, & Kendler, 2016).

Both depression and anxiety are moderately heritable disorders, with heritability estimates of 30–40% (Sullivan, Neale, & Kendler, 2000) and 20–60% (Polderman et al., 2015), respectively. Genome-wide association studies (GWAS) of depression and anxiety have largely been performed separately and without explicitly accounting for the comorbidity (Giannakopoulou, Lin, Meng, & Su, 2021; Howard et al., 2019; Levey et al., 2020, 2021; Purves et al., 2020). Given the high rate of comorbidity between depression and anxiety, these GWAS likely capture shared genetic liability for the two disorders, but may also capture distinct genetic risk factors of each disorder. Prior studies show a robust genetic correlation between depression and anxiety with estimates ranging from 80% to 95% (Mei et al., 2022). However, none of these studies specifically investigated the differences between the two disorders. A recent study showed substantial genetic overlap between the two but also trait-specific influences (Thorp et al., 2021). However, in the same study, polygenic risk scores (PRSs) did not demonstrate trait-specificity to depression and anxiety.

Electronic Health Record (EHR)-linked biobanks provide access to data from large collections of patients presenting for clinical care and have become a valuable resource for genetic studies. While EHR-derived data may not capture patients’ diagnoses as well as direct diagnostic interviews, EHR-based studies enable investigation of real-world diagnoses, including comorbidity between diagnoses, such as anxiety and depression. Biobanks also often include larger and more diverse cohorts than typical disease-specific case–control research samples. In this multi-ancestry study, we leveraged four EHR-linked biobanks from the USA to assess the contribution of genetic risk to the comorbidity of depression and anxiety. We first assessed whether PRSs for depression and anxiety, derived using results from large GWAS of patients of European ancestries primarily assessed in clinical research studies, were predictive of the respective disorders defined using diagnosis codes from EHR. Next, we evaluated how the genetic risk factors for depression and anxiety jointly contribute to the comorbidity of depression and anxiety.

## Methods

### Hospital-based biobanks

Data for this study were obtained from four different health care system biobanks: Mayo Clinic Biobank (MCB) linked to the Mayo Clinic hospital system (Bielinski et al., 2011); Bio*Me* linked to the Mount Sinai Health System in New York City, New York (Belbin et al., 2017); and two biobanks from the PsycheMERGE (electronic MEdical Record and GEnomics) Network (Zheutlin et al., 2019): BioVU linked to Vanderbilt University Medical Center (VUMC) in Nashville, Tennessee (Roden et al., 2008); and the Mass General Brigham (MGB) Biobank linked to the MGB hospital system in Boston, Massachusetts (Karlson, Boutin, Hoffnagle, & Allen, 2016). Patients enrolled in all four biobanks gave informed consent for use of their EHR data linked to their genetic data. Each site obtained institutional review board approval for the EHR-biobank research. The authors assert that all procedures contributing to this work comply with the ethical standards of the relevant national and institutional committees on human experimentation and with the Helsinki Declaration of 1975, as revised in 2008.

The EHR includes information on patients such as demographics, medications/prescriptions, laboratory values, billing codes from the International Classification of Diseases, 9th and 10th editions (ICD-9 and ICD-10-Clinical Modifications) (WHO & World Health Organization, 1993), and Current Procedural Terminology (CPT) codes. For the current study, structured EHR data for participants were extracted at the four sites during 2021 and included all patient data before that date (details are provided in online Supplementary Methods).

### Identifying cases with depression and anxiety

Depression and anxiety were defined using an initial list of ICD9/10-CM codes mapped to phecodes [i.e. higher order group of diagnoses, as described and validated by the Phecode map 1.2b1 (Denny et al., 2010)] available from https://phewascatalog.org/phecodes. (He et al., 2019; Wei et al., 2017) These definitions were then modified through expert curation from the authors. Depression was defined as having at least two depression-related ICD9/10 codes, using an initial list of ICD9/10-CM codes mapped to the phecode for depression (296.2 and 296.22) (He et al., 2019; Wei et al., 2017), with the addition of dysthymic disorder [ICD9:300.4; ICD10:F34.1], depressive type psychosis [ICD9:298.0], and atypical depressive disorder [ICD9:296.82]. Anxiety was defined as having at least two anxiety-related ICD9/10 codes, using an initial list of ICD9/10-CM codes mapped to phecodes for anxiety disorders, generalized anxiety disorders, social phobias and panic disorders, and phobias (300.1, 300.11, 300.12, 300.13, respectively) (He et al., 2019; Wei et al., 2017) with the addition of separation anxiety disorder [ICD9: 309.21; ICD10: F93.0] and removing hysteria (ICD9: 300.1) and overanxious disorder (ICD9: 313). The complete list of ICD9/10-CM codes is presented in online Supplementary Tables S1 and S2. Patients with only one diagnostic code were excluded from all analyses and controls were patients who had no documented ICD codes for depression and anxiety disorders. No exclusions were made for diagnoses of other psychiatric disorders.

### Genetic data

DNA samples from blood obtained from study participants were genotyped using genome-wide arrays, and the genetic data were imputed and processed for quality control at each site as described in the eMethods section. Briefly, single nucleotide polymorphisms were excluded using filters for call rate, minor allele frequency (<1%), and Hardy–Weinberg equilibrium. Individuals were excluded for missingness rate, sex errors, heterozygosity, and relatedness. The study included three ancestries: European, African, and Hispanic. MCB and MGB included only patients from European ancestries in their analyses due to sample size limitations of other ancestries. Genotype imputation was performed after the initial quality control and converted to best-guess genotypes for all markers with high-quality imputation (dosage-*R*^2^ > 0.8). All subsequent analyses were adjusted for principal components (PCs) within each ancestral group to reduce confounding by population substructure.

### Polygenic risk scores

To estimate the genetic risk for depression and anxiety, we calculated PRSs using the summary statistics from the Psychiatric Genomic Consortium (PGC) GWAS from major depressive disorder (MDD) (Howard et al., 2019) and anxiety (ANX) (Purves et al., 2020) working groups, respectively. Both GWASs were performed in European ancestry samples. The PRS calculations were performed separately for each ancestral group at each site. The PRSs were calculated using LDpred2 (Privé, Arbel, & Vilhjálmsson, 2020) for MCB, PRSice2 (Choi & O'Reilly, 2019) using a PRS-PCA approach (Coombes, Ploner, Bergen, & Biernacka, 2020) for Bio*Me* and MGB, and PRS-CS (Ge, Chen, Ni, Feng, & Smoller, 2019) for BioVU. The PRS-PCA approach was implemented in Bio*Me* and MGB to avoid the bias of searching for the best *p* value threshold with PRSice2.

### Statistical analysis

All analyses were stratified by site and ancestral group. Logistic regression was first used to assess whether each PRS (depression or anxiety) was associated with the matching EHR-defined diagnosis while adjusting for PCs. Next, we used multinomial regression to assess how well the combination of the MDD-PRS and ANX-PRS jointly predict the comorbidity of depression and anxiety by including either one PRS at a time or both MDD-PRS and ANX-PRS in the same model. The categorical outcome used in the multinomial model classified patients into one of four different groups: neither depression nor anxiety (controls), depression-only, anxiety-only, and both. Post-hoc pairwise comparisons were used to evaluate differences between groups. Effect sizes for PRS analyses were represented by odds ratios and percent of variation explained on the liability scale (assuming a 20% prevalence of both depression and anxiety in the general population). Meta-analyses within ancestry groups were performed using a fixed-effects meta-analysis with the meta R package.

## Results

**Figure 1.**
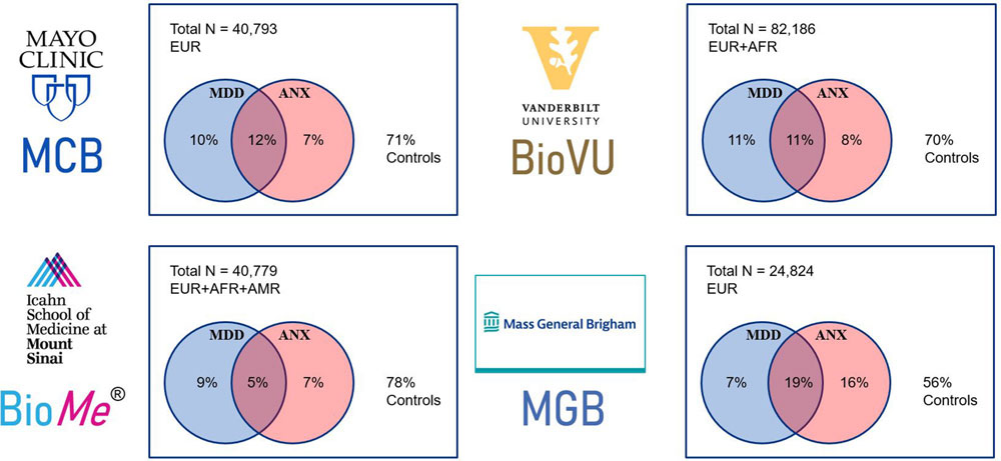
Distribution of depression (MDD) and anxiety (ANX) diagnoses at each site specified by at least two diagnosis codes from the EHR. Each site's sample size and ancestries (EUR, European; AFR, African/African American; AMR, Hispanic) are included in the top left of each site plot.

Figure 1 presents the frequencies of depression and anxiety diagnoses defined from the EHR at each site (total *N* = 177 865). Around 75% of each biobank sample had no ICD codes for depression or anxiety with the exception of MGB which had a higher prevalence of anxiety-related codes (25%) as seen in previous analyses of the data (Lee et al., 2022) and thus a lower proportion of controls. When comparing diagnostic rates across self-reported race and ethnicity in Bio*Me*, African American and Hispanic participants had higher rates of depression codes (16% and 21%, respectively) than self-identified White participants (12%). The full demographic characteristics and EHR summaries by site are shown in online Supplementary Table S3.

### PRS prediction of depression and anxiety separately

**Figure 2.**
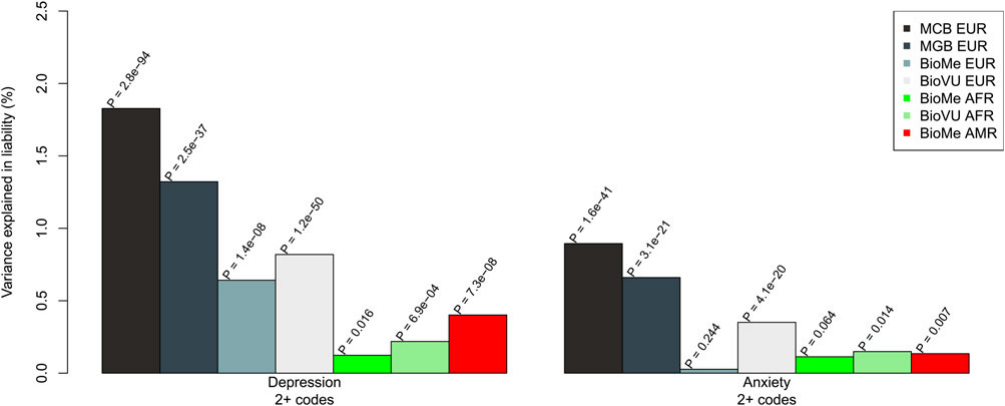
PRS prediction of depression and anxiety separately. Site- and ancestry-specific association of MDD-PRS and ANX-PRS with MDD and ANX, respectively, defined by having at least two ICD codes from the EHR. Performance is measured by variance explained by the PRS on the liability scale (assuming 20% population prevalence for both disorders). *p* values for each association are listed above each bar.

We first tested whether the MDD-PRS and ANX-PRS, trained on samples from European ancestries that are largely clinically ascertained, were associated with depression and anxiety diagnoses in the EHR, respectively. Figure 2 shows the PRS predictive performance for each site and ancestry group as measured by the percent variation explained on the liability scale. Among those of European ancestry, the MDD-PRS explained a significant proportion of variation in the liability of depression (0.6–1.8% across sites). While the MDD-PRS was also associated with depression in the African American and Hispanic participants from BioVU and Bio*Me*, the predictive performance was markedly attenuated, explaining only 0.10–0.40% of variation in the liability of depression.

In comparison to the performance of the MDD-PRS, the ANX-PRS explained less variation in the liability of anxiety in European cohorts (0–0.9% across sites) and was not predictive of anxiety in Bio*Me* (*p* = 0.24). Similar to MDD-PRS, the performance of the ANX-PRS was reduced in the non-European cohorts and was only significant in the BioVU African American sample (*R*^2^ = 0.19%; *p* = 0.014). The full results are shown in online Supplementary Table S4.

### Joint PRS prediction of depression and anxiety comorbidity

Next, we tested whether both PRSs were associated with the comorbidity of depression and anxiety using a multinomial outcome specifying whether a participant had neither diagnosis (*N* = 132 960), depression-only (*N* = 16 092), anxiety-only (*N* = 13 098), or comorbid (*N* = 16 584). In the combined meta-analysis across participants of European ancestry at all sites (total *N* = 138 632), both MDD-PRS and ANX-PRS were significantly associated with each case subgroup compared to controls after adjusting for one another (Table 1). Notably, both the MDD-PRS (OR 1.26 per s.d. increase in PRS; 95% CI 1.24–1.29; *p* = 1 × 10^−105^) and the ANX-PRS (OR 1.09; 95% CI 1.07–1.11; *p* = 3 × 10^−15^) had the largest effect size in the comorbid group when compared to controls after adjusting for the other PRS. Furthermore, the MDD-PRS was significantly higher in the comorbid group than the depression-only group (OR 1.09; 95% CI 1.06–1.12; *p* = 7 × 10^−8^) and the anxiety-only group (OR 1.15; 95% CI 1.11–1.19; *p*<1 × 10^−16^) and was significantly higher in the depression-only group than the anxiety-only group (OR 1.06; 95% CI 1.02–1.09; *p* = 0.001), showing a gradient of genetic risk across the isolated conditions and the comorbidity (online Supplementary Table S6).

**Table 1.** Joint PRS prediction of depression and anxiety comorbidity from the multinomial model

Site-ancestry (*N* control)	Group	*N* case	MDD-PRS OR (95% CI)	MDD-PRS *p* value	ANX-PRS OR (95% CI)	ANX-PRS *p* value
Meta-EUR (*N* = 102 113)	Depression-only	12 201	1.16 (1.14, 1.19)	3 × 10^−39^	1.04 (1.01, 1.06)	0.001
	Anxiety-only	10 299	1.10 (1.07, 1.13)	1 × 10^−14^	1.07 (1.04, 1.09)	3 × 10^−7^
	Comorbid	14 019	1.26 (1.24, 1.29)	1 × 10^−105^	1.09 (1.07, 1.11)	3 × 10^−15^
Meta-AFR (*N* = 20 810)	Depression-only	2339	1.06 (0.99, 1.13)	0.117	1.00 (0.93, 1.07)	0.98
	Anxiety-only	1064	1.01 (0.93, 1.10)	0.807	1.02 (0.94, 1.11)	0.64
	Comorbid	1399	1.13 (1.04, 1.23)	0.005	1.05 (0.97, 1.14)	0.25
Bio*Me*-AMR (*N* = 10 037)	Depression-only	1552	1.34 (0.92, 1.95)	0.125	0.68 (0.49, 0.93)	0.015
	Anxiety-only	689	1.36 (1.05, 1.77)	0.02	0.80 (0.65, 1.00)	0.052
	Comorbid	1343	2.12 (1.61, 2.80)	1 × 10^−7^	0.87 (0.69, 1.10)	0.26

EUR, European ancestry; AFR, African ancestry; AMR, Hispanic ancestry; OR, odds ratio associated with 1 s.d. increase in the PRS; PRS, polygenic risk score; MDD, major depressive disorder; ANX, anxiety-related disorders.

In the meta-analysis of African American participants from Bio*Me* and BioVU (*N* = 25 612), after adjusting for the contribution of the ANX-PRS, the MDD-PRS was associated only with the comorbid (*N* = 1399; OR 1.13; 95% CI 1.04–1.23; *p* = 0.005) group when compared to controls (*N* = 20 810), and not with depression-only (*N* = 2339; OR 1.06; 95% CI 0.99–1.13; *p* = 0.12) and ANX-only (*N* = 1064; OR 1.01; 95% CI 0.93–1.10; *p* = 0.81). Among the Hispanic participants from Bio*Me* (*N* = 9034), the MDD-PRS was only predictive of the comorbid group (*N* = 1343; OR 2.12; 95% CI 1.61–2.80; *p* = 1 × 10^−7^) compared to controls (*N* = 10 037). After adjusting for the contribution of MDD-PRS, the ANX-PRS was not predictive of any case subgroup compared to controls among African American and Hispanic participants. The full results by ancestry and by site are presented in online Supplementary Tables S5 and 6.

## Discussion

Prior GWASs of depression and anxiety disorders typically have not considered the common comorbidity between depression and anxiety. Therefore, little is known about how genetic risk impacts the comorbidity. To study this, we performed a multi-site and multi-ancestry PRS analysis across four different EHR-linked biobanks using genetic risk of depression and anxiety. The MDD-PRS predicted diagnosis of depression in the EHR in all ancestries and at all sites. However, ANX-PRS was less predictive of anxiety diagnoses, with the ANX-PRS having a smaller effect size than MDD-PRS and showing no significant associations with anxiety disorders among any ancestries in Bio*Me*. Importantly though, in our model of the comorbidity in European ancestry samples, the MDD-PRS showed a gradient of risk with respect to diagnosis of depression and anxiety such that patients with the comorbidity had the highest MDD-PRS followed by those with depression-only, anxiety-only, and neither diagnosis. Although not statistically significant, anxiety genetic risk showed a similar trend with the comorbid group having the highest genetic risk for anxiety followed by those with anxiety-only, depression-only, and neither. The observed gradient of genetic risk across the diagnostic groups, with the MDD-PRS being significantly higher for patients with depression-only than those with anxiety-only after adjusting for ANX-PRS, suggests that the additive genetic factors may partially differ between the two disorders.

We found that the comorbid group was at the highest level of genetic risk for depression and anxiety, which could indicate that genetic factors play a larger role in those with the comorbidity compared with only one diagnosed disorder. However, it is important to consider how our study design may also have impacted these results. In our study, depression and anxiety were defined with ICD codes that have only modest concordance with cases identified by structured or semi-structured clinical assessments used in research (Fiest et al., 2014). Thus, while our finding that the MDD + ANX group has the highest genetic risk of MDD could reflect true biological differences, it might instead reflect the fact that the MDD + ANX comorbidity is more clinically conspicuous and therefore better captured in the EHR because it is associated with more severe depressive symptoms, higher levels of impairment, poorer clinical outcomes, and more suicidal ideation (Joffe et al., 1993; Nam et al., 2016; Pollack, 2005; Zhou et al., 2017). These patients may be more likely to be referred for specialty care resulting in a higher likelihood of a depression or anxiety diagnosis. If so, our PRS findings may still reflect biologically determined greater symptom severity in the MDD + ANX group. Consequently, repeating this study in a large sample with more rigorous diagnostic assessments is warranted.

A strength of this study is that a larger range of the phenome can be easily and cost-effectively captured through the EHR. While non-EHR clinical studies may have more in-depth assessment of the phenotype of interest, they typically lack comprehensive assessment of important comorbidities and have smaller sample sizes. It is also important to acknowledge the limitations of EHR-based studies; most notably, reliance on ICD codes from the EHR can result in misclassification of cases and controls. A strategy to improve case/control ascertainment from the EHR is to use natural language processing (NLP) to incorporate clinical notes into the classification system (Ford, Carroll, Smith, Scott, & Cassell, 2016). Such an approach requires NLP algorithms that translate well across health systems, which is an active area of research. Heterogeneity of diagnosis prevalence between the different sites may have also contributed to differential power in the analyses. Given this, it is perhaps more striking that the PRSs demonstrated significant association despite heterogeneity of patient populations, diagnostic practices, and differences in EHR systems across sites, albeit with small amounts of variance explained for diagnosis of depression and anxiety. Notably, the MDD-PRS was less predictive of EHR diagnosis of MDD in the EHR (liability-*R*^2^ ranging from 0.1% to 1.8%) than that seen in out-of-sample prediction in research cohorts (liability-*R*^2^ ranging from 1.5% to 3.2%) (Howard et al., 2019) whereas the prediction performance of ANX-PRS for EHR diagnosis of anxiety was similar to that seen in the UK Biobank, ANGST, and iPSYCH (liability-*R*^2^ ranging from 0.1% to 0.7%) (Purves et al., 2020).

Finally, this study of depression and anxiety was one of the first to include samples from ancestrally diverse groups. However, the sample sizes of African and Hispanic ancestry patients were much smaller than the European ancestry group, resulting in lower power to detect associations with genetic risk. Furthermore, the constructed PRSs were based on cohorts with European ancestry, which are known to have poorer prediction in non-European ancestries (Martin et al., 2019). The African American and Hispanic patients also had higher rates of depression diagnoses which is contrary to what might be expected based on prior epidemiological (Breslau, Kendler, Su, Gaxiola-Aguilar, & Kessler, 2005) or clinical (Stockdale, Lagomasino, Siddique, McGuire, & Miranda, 2008) research. Moreover, diagnostic biases (Gara et al., 2012) and decreased healthcare access for people from marginalized communities (Bailey, Mokonogho, & Kumar, 2019) can contribute to the misclassification in these groups and thus further reduce power.

In our study, genetic risks of depression and anxiety contributed jointly to depression and anxiety, but each PRS showed stronger association with the corresponding diagnosis. Our results suggest that depression and anxiety may have partially independent genetic liabilities and the genetic vulnerabilities to depression and anxiety make distinct contributions to comorbid depression and anxiety. It is also important to note that the prior GWASs of MDD and anxiety from which the PRSs were computed did not exclude individuals with comorbid anxiety and depression and thus, it is expected that both the depression and anxiety PRSs also captured the shared liability limiting the unique contributions from these PRS to the comorbid condition. Future large separate GWASs of depression, anxiety, and their comorbidity are needed to fully explore the extent to which anxiety and depression have distinct liabilities. Nevertheless, even without these GWAS to create comorbid-specific PRS, we found that the MDD-PRS and ANX-PRS were independently associated with the comorbidity of depression and anxiety in the EHR, supporting the hypothesis that the correlated disorders represent partially distinct nosological entities.

## Supporting information

Coombes et al. supplementary material Coombes et al. supplementary material

## Supplementary material

The supplementary material for this article can be found at https://doi.org/10.1017/S0033291723000983.
