# Supplementary material for: The genetic contribution to the comorbidity of depression and anxiety: a multi-site electronic health records study of almost 178 000 people
Source: Psychol Med. 2023 May 5;53(15):7368–74. doi: 10.1017/S0033291723000983 (PMC10719682; doi:10.1017/S0033291723000983)
Supplement: Coombes et al. supplementary material [file S0033291723000983sup001.docx]

The contribution of genetic risk to the comorbidity of depression and anxiety: a multi-site electronic health records study

**Online Supplementary Material**

#

# **Cohort Descriptions**

## ***Mayo Clinic Biobank***

Cases and controls were ascertained using EHR data from 57,001 patients from the Mayo Clinic Biobank. EHR data for the participants was extracted on September 23, 2022, and included any diagnostics on or before April 6, 2020, the date patient consent was checked. The Institutional Review Board of Mayo Clinic approved this study. Samples were sequenced at the Regeneron Genetics Center (RGC) using a custom design that additionally augments the exome capture with “backbone” regions intended to measure common tagging variation for purposes of GWAS. These backbone regions are targeted at lower depth and undergo substantial post-processing that can boost genotyping quality based on shared information via linkage disequilibrium and population allele frequencies. This is referred to by RGC as genotyping-by-sequencing (GxS).

The resulting GxS data was run through the Mayo Clinic Genotype QC pipeline. In this QC pipeline, SNPs were excluded using filters for call rate (<95%), minor allele frequency (<0.5%), and Hardy-Weinberg Equilibrium (p < 1e-6). Individuals were excluded for excessive missing data (>5%), sex errors, abnormal heterozygosity (< 70% on multiple chromosomes). Analysis of genetic ancestry was performed on a subset of 4874 high-quality HapMap3 SNPs. Principal components analysis (PCA) was first performed on the HapMap3 samples and then MCB samples were projected onto these PCs. Kernel density estimators were trained for each of the individual HapMap3 populations and mapped to one of five main ancestral super-populations (AFR=African; AMR=Admixed American; EAS=East Asian; EUR=European; SAS=South Asian) based on specific likelihood criteria for the individual populations such that the likelihood for a given ancestry group was greater than 0.3, the sample was assigned to that ancestry group. When two ancestry groups had a likelihood of greater than 0.3, RGC assigned AFR over EUR, AMR over EUR, AMR over EAS, SAS over EUR, and AMR over AFR.

Cryptic relatedness analysis was performed in an iterative process using PLINK and PRIMUS to estimate IBD sharing. Highly related samples were removed from the sample if they had >100 closely related samples (PI_HAT>0.1875) or >25000 related samples (PI_HAT>0.08); the relatedness analysis was performed iteratively until no such samples remained. For each pair with a estimated 2^nd^ degree or higher relatedness, we removed the individual with shorter length of EHR record.

## ***BioMe***

Cases and controls were ascertained using EHR data from 48,718 patients from the Bio*Me* biobank in Mount Sinai Health System[^1^](https://nam12.safelinks.protection.outlook.com/?url=https%3A%2F%2Furldefense.proofpoint.com%2Fv2%2Furl%3Fu%3Dhttps-3A__paperpile.com_c_JX9KZN_nzmc%26d%3DDwMGaQ%26c%3DshNJtf5dKgNcPZ6Yh64b-ALLUrcfR-4CCQkZVKC8w3o%26r%3DNw5cDZTYDLN00aCTXxQMm11yFMnMnizzrBA4IRTSrnk%26m%3DwtlK3SmMvNLNAGvWAlFem25h2CVeBRhLPcBWGATHdmfihKgWiTTS2wcjOdzbJvpU%26s%3Ds3DQ_F3SJgAOiBYhP4r5t25a_0DI3LP4TXxC1JlkCa4%26e%3D&data=05%7C01%7CCoombes.Brandon%40mayo.edu%7C8c503de7e6974ab0e25d08dac3316cea%7Ca25fff9c3f634fb29a8ad9bdd0321f9a%7C0%7C0%7C638036914410243284%7CUnknown%7CTWFpbGZsb3d8eyJWIjoiMC4wLjAwMDAiLCJQIjoiV2luMzIiLCJBTiI6Ik1haWwiLCJXVCI6Mn0%3D%7C3000%7C%7C%7C&sdata=mLJcWQY%2FElATHQjGvQmjRJzTffvQk0ZKEQMvgeuOiak%3D&reserved=0). EHR data for the participants was extracted in September 2022 and included any diagnostics on or before that date. DNA for GWAS analysis was isolated from whole blood and genotyped in two freezes, using the Illumina Global Screening Array (GSA) and the Illumina Global Diversity Array (GDA) platforms, respectively. The Institutional Review Board approved this study. QC and imputation have been described previously.[^3^](https://nam12.safelinks.protection.outlook.com/?url=https%3A%2F%2Furldefense.proofpoint.com%2Fv2%2Furl%3Fu%3Dhttps-3A__paperpile.com_c_JX9KZN_RBDW%26d%3DDwMGaQ%26c%3DshNJtf5dKgNcPZ6Yh64b-ALLUrcfR-4CCQkZVKC8w3o%26r%3DNw5cDZTYDLN00aCTXxQMm11yFMnMnizzrBA4IRTSrnk%26m%3DwtlK3SmMvNLNAGvWAlFem25h2CVeBRhLPcBWGATHdmfihKgWiTTS2wcjOdzbJvpU%26s%3D72F6e87r6FUWc3b7JXyDc0fZDVC4yND86n7Cb7ev4jg%26e%3D&data=05%7C01%7CCoombes.Brandon%40mayo.edu%7C8c503de7e6974ab0e25d08dac3316cea%7Ca25fff9c3f634fb29a8ad9bdd0321f9a%7C0%7C0%7C638036914410243284%7CUnknown%7CTWFpbGZsb3d8eyJWIjoiMC4wLjAwMDAiLCJQIjoiV2luMzIiLCJBTiI6Ik1haWwiLCJXVCI6Mn0%3D%7C3000%7C%7C%7C&sdata=oVR8Na8wV02T%2Fy2a6MD2eUgnrV7R0UrlgJ9940xgIXM%3D&reserved=0) Briefly, in the QC pipeline, SNPs were excluded using filters for call rate (<95%), minor allele frequency (<1%), and Hardy-Weinberg Equilibrium (p < 1e-8). Individuals were excluded for excessive missing data (>5%), sex errors, abnormal heterozygosity (±3 SD for each ancestry and combined) and relatedness (removing a random individual from any pair with kinship coefficient > 0.2). Imputation was conducted with Michigan Imputation Server 1000G P3. For the combined set (GDA and GSA) we adapted the merging strategy from eMERGE ([https://www.frontiersin.org/articles/10.3389/fgene.2014.00370/full](https://nam12.safelinks.protection.outlook.com/?url=https%3A%2F%2Fwww.frontiersin.org%2Farticles%2F10.3389%2Ffgene.2014.00370%2Ffull&data=05%7C01%7CCoombes.Brandon%40mayo.edu%7C8c503de7e6974ab0e25d08dac3316cea%7Ca25fff9c3f634fb29a8ad9bdd0321f9a%7C0%7C0%7C638036914410243284%7CUnknown%7CTWFpbGZsb3d8eyJWIjoiMC4wLjAwMDAiLCJQIjoiV2luMzIiLCJBTiI6Ik1haWwiLCJXVCI6Mn0%3D%7C3000%7C%7C%7C&sdata=%2F0kay5rXpol5re3f8qf4UJxvj4zch4etaCZaCaD%2FKnA%3D&reserved=0)). Imputed datasets were merged based on the set of intersecting markers [only markers that were of high quality in all of the imputed data were combined (i.e., info score >0.7)]. Duplicate samples were removed, whereby the highest quality version of the sample was maintained.

## ***BioVU***

## Cases and controls were ascertained using EHR data from 82186 patients from the Vanderbilt University Medical Center (VUMC)[^5^](https://paperpile.com/c/JX9KZN/X2Y2). EHR data for the participants was extracted in June 2021 and included any diagnostics on or before that date. DNA for GWAS analysis was isolated from whole blood and genotyped using the Illumina MEGAEX platform. The VUMC Institutional Review Board approved this study. In the QC pipeline, SNPs were excluded using filters for call rate (<2%), minor allele frequency (<1%), and Hardy-Weinberg Equilibrium (p < 5e-5). Individuals were excluded for excessive missing data (>2%), sex errors, abnormal heterozygosity (|Fhet| > 0.2) and relatedness (removing a random individual from any pair with kinship coefficient > 0.2). After QC, genotypes were imputed using SHAPEIT[^6^](https://paperpile.com/c/JX9KZN/Ie9z)/IMPUTE4[^4^](https://paperpile.com/c/JX9KZN/pHcX) with the 1000 genomes phase I reference panel. Genotype imputation was performed after the initial QC and converted to best-guess genotypes for all markers with high-quality imputation (INFO > 0.3).

## ***Partners Biobank (MGB)***

Cases and controls were ascertained using EHR data from 24,842 patients from the Mass General Brigham (MGB) Health System. EHR data for the participants was extracted in February 2020 and included any diagnoses on or before that date. DNA for GWAS analysis was isolated from blood and genotyped using Illumina arrays (MEGA, MEGAEX, and MEG BeadChip). The Institutional Review Board approved this study. In the QC pipeline, SNPs were excluded using filters for call rate (<95%), and Hardy-Weinberg Equilibrium (p<1e-10). Individuals were excluded for excessive missing data (>2%), sex errors, abnormal heterozygosity (±3 SD) and relatedness (removing a random individual from any pair with kinship coefficient > 0.2). After QC, batches from genotyping arrays were merged and then imputed using the Michigan Imputation server (Minimac4 1.2.1) with the HRC/1KG reference panel (Reference Panel: apps@hrc-r1.1 [hg19])[^2^](https://paperpile.com/c/JX9KZN/uL5f). Genotype imputation was performed after the initial QC and converted to best-guess genotypes for all markers with high-quality imputation (dosage-R2 > 0.8) and common minor allele frequency (>1%).

# **Supplemental Table 1.** ICD 9/10 codes used for defining depression with prevalence listed among Mayo Clinic Biobank and Bio*Me*.

| **ICD9/10** | **ICD** | **Phecode** | **Description** | **MCB**  N=41K | **Bio*Me***  N=56K |
| --- | --- | --- | --- | --- | --- |
| 296.2 | ICD9 | 296.22 | Major depressive disorder, single episode | 0.0% | - |
| 296.2 | ICD9 | 296.22 | Major depressive disorder, single episode, unspecified degree | 6.0% | - |
| 296.21 | ICD9 | 296.2 | Major depressive disorder, single episode, mild degree | 0.9% | - |
| 296.22 | ICD9 | 296.22 | Major depressive disorder, single episode, moderate degree | 1.7% | - |
| 296.23 | ICD9 | 296.22 | Major depressive disorder, single episode, severe degree, w/o psychosis | 0.5% | - |
| 296.24 | ICD9 | 296.22 | Major depressive disorder, single episode, severe degree, spec. psychosis | 0.1% | - |
| 296.25 | ICD9 | 296.22 | Major depressive disorder, single episode, in partial or unspec. remission | 1.0% | - |
| 296.26 | ICD9 | 296.22 | Major depressive disorder, single episode in full remission | 1.2% | - |
| 296.3 | ICD9 | 296.22 | Major depressive disorder, recurrent episode | 0.0% | - |
| 296.3 | ICD9 | 296.22 | Major depressive disorder, recurrent episode, unspecified degree | 2.8% | - |
| 296.31 | ICD9 | 296.2 | Major depressive disorder, recurrent episode, mild degree | 2.5% | - |
| 296.32 | ICD9 | 296.22 | Major depressive disorder, recurrent episode, moderate degree | 4.1% | - |
| 296.33 | ICD9 | 296.22 | Major depressive disorder, recurrent episode, severe degree, wo psychosis | 1.7% | - |
| 296.34 | ICD9 | 296.22 | Major depressive disorder, rec episode, severe degree, spec. psychosis | 0.2% | - |
| 296.35 | ICD9 | 296.22 | Major depressive disorder, recurrent episode, in partial/unspec. remission | 2.3% | - |
| 296.36 | ICD9 | 296.22 | Major depressive disorder, recurrent episode, in full remission | 2.3% | - |
| 311 | ICD9 | 296.2 | Depressive disorder NEC | 18.1% | - |
| 300.4 | ICD9 | 300.4 | Dysthymic disorder | 6.9% | - |
| 298.0 | ICD9 | 295.3 | Depressive type psychosis | 0.0% | - |
| 296.82 | ICD9 | 296.1 | Atypical depressive disorder | 0.0% | - |
| F32 | ICD10 | 296.22 | Major depressive disorder, single episode | 0.0% | 0.6% |
| F32.0 | ICD10 | 296.22 | Major depressive disorder, single episode, mild | 0.8% | 0.6% |
| F32.1 | ICD10 | 296.22 | Major depressive disorder, single episode, moderate | 1.4% | 0.7% |
| F32.2 | ICD10 | 296.22 | Major depressive disorder, single episode, severe w/o psychotic features | 0.4% | 0.2% |
| F32.3 | ICD10 | 296.22 | Major depressive disorder, single episode, severe with psychotic features | 0.0% | 0.1% |
| F32.4 | ICD10 | 296.22 | Major depressive disorder, single episode, in partial remission | 0.5% | 0.1% |
| F32.5 | ICD10 | 296.22 | Major depressive disorder, single episode, in full remission | 1.1% | 0.3% |
| F32.8 | ICD10 | 296.22 | Other depressive episodes | 0.1% | - |
| F32.89 | ICD10 | 296.22 | Single episode of 'masked' depression NOS | 0.0% | - |
| F32.9 | ICD10 | 296.22 | Major depressive disorder, single episode, unspecified | 13.8% | 13.7% |
| F33 | ICD10 | 296.22 | Major depressive disorder, recurrent | 3.1% | 0.6% |
| F33.0 | ICD10 | 296.22 | Major depressive disorder, recurrent, mild | 0.0% | 0.6% |
| F33.1 | ICD10 | 296.22 | Major depressive disorder, recurrent, moderate | 4.7% | 1.9% |
| F33.2 | ICD10 | 296.22 | Major depressive disorder, recurrent severe without psychotic features | 1.6% | 0.4% |
| F33.3 | ICD10 | 296.22 | Major depressive disorder, recurrent, severe with psychotic symptoms | 0.1% | 0.2% |
| F33.4 | ICD10 | 296.22 | Major depressive disorder, recurrent, in remission | 0.4% | 0.1% |
| F33.40 | ICD10 | 296.22 | Major depressive disorder, recurrent, in remission, unspecified | - | - |
| F33.41 | ICD10 | 296.22 | Major depressive disorder, recurrent, in partial remission | 2.2% | 0.5% |
| F33.42 | ICD10 | 296.22 | Major depressive disorder, recurrent, in full remission | 2.9% | 0.6% |
| F33.8 | ICD10 | 296.22 | Other recurrent depressive disorders | 0.0% | 0.0% |
| F33.9 | ICD10 | 296.22 | Major depressive disorder, recurrent, unspecified | 2.2% | 0.9% |
| F34.1 | ICD10 | 300.4 | Dysthymic disorder | 1.5% | 1.3% |

# **Supplemental Table 2.** ICD 9/10 codes used for defining anxiety with prevalence listed among Mayo Clinic Biobank and Bio*Me*.

| **ICD9/10** | **ICD** | **PheCode** | **ICD9 String** | **MCB**  N=41K | **Bio*Me***  N=56K |
| --- | --- | --- | --- | --- | --- |
| 293.84 | ICD9 | 300.1 | Anxiety disorder in conditions classified elsewhere | 0.6% | - |
| 300 | ICD9 | 300.1 | Anxiety states | 15.6% | - |
| 300 | ICD9 | 300.1 | Anxiety state unspecified | 0.0% | - |
| 300.01 | ICD9 | 300.12 | Panic disorder without agoraphobia | 1.6% | - |
| 300.02 | ICD9 | 300.11 | Generalized anxiety disorder | 4.2% | - |
| 300.09 | ICD9 | 300.1 | Other anxiety states | 0.2% | - |
| 300.2 | ICD9 | 300.13 | Phobia unspecified | 0.0% | - |
| 300.21 | ICD9 | 300.12 | Agoraphobia with panic disorder | 0.5% | - |
| 300.22 | ICD9 | 300.12 | Agoraphobia without mention of panic attacks | 0.1% | - |
| 300.23 | ICD9 | 300.12 | Social phobia | 0.4% | - |
| 300.29 | ICD9 | 300.13 | Other isolated or specific phobias | 0.4% | - |
| 309.21 | ICD9 | 313 | Separation anxiety disorder | 0.0% | - |
| F06.4 | ICD10 | 300.1 | Organic anxiety disorder | 0.4% | 0.1% |
| F40 | ICD10 | 300.13 | Phobic anxiety disorders | 0.0% | 0.0% |
| F40.0 | ICD10 | 300.12 | Agoraphobia | 0.2% | 0.2% |
| F40.1 | ICD10 | 300.12 | Social phobias | 0.2% | 0.1% |
| F40.2 | ICD10 | 300.13 | Specific (isolated) phobias | 0.3% | 0.1% |
| F40.8 | ICD10 | 300.13 | Other phobic anxiety disorders | - | 0.0% |
| F40.9 | ICD10 | 300.13 | Phobic anxiety disorder, unspecified | 0.0% | 0.0% |
| F41.0 | ICD10 | 300.12 | Panic disorder [episodic paroxysmal anxiety] | 0.7% | 1.1% |
| F41.1 | ICD10 | 300.11 | Generalized anxiety disorder | 4.7% | 3.1% |
| F41.2 | ICD10 | 300.1 | Mixed anxiety and depressive disorder | - | - |
| F41.3 | ICD10 | 300.1 | Other mixed anxiety disorders | 0.0% | 0.0% |
| F41.8 | ICD10 | 300.1 | Other specified anxiety disorders | 4.3% | 1.5% |
| F41.9 | ICD10 | 300.1 | Anxiety disorder, unspecified | 10.2% | 12.6% |

**Supplemental Table 3**. Demographic and EHR summary statistics across site.

|  | **Site** | **MCB** | **MGB** | **Bio*Me*** | **Bio*Me*** | **Bio*Me*** | **BioVU** | **BioVU** |
| --- | --- | --- | --- | --- | --- | --- | --- | --- |
|  | **Ancestry** | **EUR** | **EUR** | **EUR** | **AFR** | **AMR** | **EUR** | **AFR** |
| **Variable** | **N** | 40793 | 21049 | 15828 | 11330 | 13621 | 60962 | 14282 |
|  | Control | 28869 (70.8%) | 14208 (67.5%) | 12964 (81.9%) | 9076 (80.1%) | 10037 (73.7%) | 46072 (75.6%) | 11734 (82.2%) |
| Diagnosis group | MDD-only | 4138 (10.1%) | 1297 (6.2%) | 993 (6.3%) | 1162 (10.3%) | 1552 (11.4%) | 5773 (9.5%) | 1177 (8.2%) |
|  | ANX-only | 2857 (7%) | 2249 (10.7%) | 1023 (6.5%) | 421 (3.7%) | 689 (5.1%) | 4170 (6.8%) | 643 (4.5%) |
|  | MDD+ANX | 4929 (12.1%) | 3295 (15.7%) | 848 (5.4%) | 671 (5.9%) | 1343 (9.9%) | 4947 (8.1%) | 728 (5.1%) |
| Current Age (yrs) | Mean (SD) | 70.5 (14.8) | 62.89 (16.31) | 60.4 (19.5) | 60.2 (15.5) | 61.2 (16.6) | 53 (22.6) | 40 (21.3) |
| Female | N (%) | 26091 (58.4%) | 13228 (53%) | 5148 (50.7%) | 4302 (61.1%) | 5511 (61%) | 39569 (59.1) | 9,032 (59.1) |
| EHR length (yrs) | Mean (SD) | 18.3 (11.3) | 13.0 (7.2) | 9.8 (2.1) | 10.2 (2.3) | 10.2 (2.8) | 10.7 (6.97) | 10.3 (7.3) |
| Total ICD codes | Mean (SD) | 445.0 (581.5) | 611.1 (741.4) | 126.2 (222.8) | 243.7 (344.9) | 238.7 (350.5) | 278.9 (390.8) | 255.5 (419.7) |

**Supplemental Table 4. PRS prediction of depression and anxiety separately.** Results are show for depression (MDD) and anxiety (ANX) defined from EHR with 2+ codes required. R^2^ = percent variation explained on the liability scale assuming 20% prevalence of both depressive and anxiety disorders.

|  | **Site** | **Ancestry** | **Ncont** | **Ncase** | **OR** | **R^2^ liability** | **pval** |
| --- | --- | --- | --- | --- | --- | --- | --- |
| MDD | MCB | EUR | 32889 | 9993 | 1.28 | 1.8% | 3E-94 |
|  | MGB | EUR | 18077 | 5250 | 1.23 | 1.3% | 3E-37 |
|  | BioMe | EUR | 14595 | 1841 | 1.94 | 0.6% | 1E-08 |
|  | BioVU | EUR | 51644 | 10720 | 1.17 | 0.8% | 1E-50 |
|  | BioMe | AFR | 9825 | 1833 | 1.36 | 0.1% | 0.016 |
|  | BioVU | AFR | 12607 | 1905 | 1.09 | 0.2% | 0.00070 |
|  | BioMe | AMR | 11228 | 2895 | 1.72 | 0.4% | 7E-08 |
| ANX | MCB | EUR | 34107 | 8271 | 1.19 | 0.9% | 2E-41 |
|  | MGB | EUR | 16130 | 6108 | 1.16 | 0.7% | 3E-21 |
|  | BioMe | EUR | 14359 | 1871 | 0.9 | 0.0% | 0.24 |
|  | BioVU | EUR | 53373 | 9117 | 1.11 | 0.4% | 4E-20 |
|  | BioMe | AFR | 10765 | 1092 | 0.75 | 0.1% | 0.064 |
|  | BioVU | AFR | 13206 | 1371 | 1.07 | 0.2% | 0.014 |
|  | BioMe | AMR | 12144 | 2032 | 0.77 | 0.1% | 0.0070 |

**Supplemental Table 5. PRS prediction of depression and anxiety versus controls.** Results are show for PRSs for depression (MDD) and anxiety (ANX) predicting anxiety and depression comorbid group compared to controls defined from EHR with 2+ codes required. Highlight indicates p-value < 0.01.

|  |  |  | **Models including both PRS** | | | | **Models including one PRS at a time** | | | |
| --- | --- | --- | --- | --- | --- | --- | --- | --- | --- | --- |
| **Site-Ancestry**  **(N control)** | **Case group** | **N case** | **MDD-PRS**  **OR (95% CI)** | **p-value** | **ANX-PRS**  **OR (95% CI)** | **p-value** | **MDD-PRS**  **OR (95% CI)** | **p-value** | **ANX-PRS**  **OR (95% CI)** | **p-value** |
| META-EUR  (N = 102,113) | MDD-only | 12201 | 1.16 (1.14, 1.19) | 3E-39 | 1.04 (1.01, 1.06) | 0.001 | 1.18 (1.16, 1.20) | 1E-59 | 1.10 (1.08, 1.13) | 6E-22 |
|  | Anxiety-only | 10299 | 1.10 (1.07, 1.13) | 1E-14 | 1.07 (1.04, 1.09) | 3E-07 | 1.13 (1.11, 1.16) | 4E-29 | 1.11 (1.08, 1.13) | 2E-20 |
|  | Comorbid | 14019 | 1.26 (1.24, 1.29) | 1E-105 | 1.09 (1.07, 1.11) | 3E-15 | 1.31 (1.28, 1.33) | 2E-170 | 1.20 (1.18, 1.22) | 1E-78 |
| META-AFR  (N = 20,810) | MDD-only | 2339 | 1.06 (0.99, 1.13) | 0.117 | 1.00 (0.93, 1.07) | 0.98 | 1.07 (1.01, 1.14) | 0.030 | 1.03 (0.97, 1.09) | 0.37 |
|  | Anxiety-only | 1064 | 1.01 (0.93, 1.10) | 0.807 | 1.02 (0.94, 1.11) | 0.64 | 1.04 (0.96, 1.12) | 0.30 | 1.03 (0.95, 1.11) | 0.47 |
|  | Comorbid | 1399 | 1.13 (1.04, 1.23) | 0.005 | 1.05 (0.97, 1.14) | 0.25 | 1.17 (1.08, 1.26) | 5E-05 | 1.09 (1.01, 1.17) | 0.019 |
| BioMe-AMR  (N = 10,037) | MDD-only | 1552 | 1.34 (0.92, 1.95) | 0.125 | 0.68 (0.49, 0.93) | 0.015 | 1.21 (0.91, 1.61) | 0.19 | 0.68 (0.53, 0.87) | 0.002 |
|  | Anxiety-only | 689 | 1.36 (1.05, 1.77) | 0.02 | 0.80 (0.65, 1.00) | 0.052 | 1.39 (1.10, 1.74) | 0.005 | 0.76 (0.63, 0.92) | 0.006 |
|  | Comorbid | 1343 | 2.12 (1.61, 2.80) | 1E-07 | 0.87 (0.69, 1.10) | 0.26 | 2.04 (1.62, 2.57) | 2E-09 | 0.73 (0.60, 0.89) | 0.002 |
| MCB-EUR  (N = 28,869) | MDD-only | 4138 | 1.20 (1.16, 1.25) | < 1E-16 | 1.00 (0.96, 1.04) | 0.96 | 1.20 (1.16, 1.24) | < 1E-16 | 1.08 (1.05, 1.12) | 5E-06 |
|  | Anxiety-only | 2857 | 1.12 (1.07, 1.17) | 9E-07 | 1.08 (1.03, 1.12) | 0.001 | 1.15 (1.11, 1.20) | 3E-12 | 1.13 (1.08, 1.17) | 3E-09 |
|  | Comorbid | 4929 | 1.32 (1.28, 1.37) | < 1E-16 | 1.11 (1.07, 1.15) | 6E-09 | 1.38 (1.34, 1.43) | < 1E-16 | 1.25 (1.21, 1.29) | < 1E-16 |
| MGB-EUR  (N = 14,208) | MDD-only | 1297 | 1.17 (1.10, 1.24) | 9E-07 | 1.05 (0.98, 1.11) | 0.15 | 1.19 (1.12, 1.26) | 4E-09 | 1.11 (1.05, 1.17) | 4E-04 |
|  | Anxiety-only | 2249 | 1.08 (1.03, 1.14) | 0.002 | 1.09 (1.03, 1.14) | 0.001 | 1.12 (1.07, 1.17) | 2E-06 | 1.12 (1.07, 1.17) | 1E-06 |
|  | Comorbid | 3295 | 1.25 (1.19, 1.30) | < 1E-16 | 1.10 (1.06, 1.15) | 5E-06 | 1.29 (1.24, 1.34) | < 1E-16 | 1.20 (1.15, 1.25) | < 1E-16 |
| BioMe  (N = 12,964) | MDD-only | 993 | 2.49 (1.83, 3.37) | 5E-09 | 0.91 (0.72, 1.16) | 0.44 | 2.26 (1.77, 2.88) | 5E-11 | 0.80 (0.66, 0.97) | 0.025 |
|  | Anxiety-only | 1023 | 1.72 (1.27, 2.35) | 6E-04 | 0.73 (0.57, 0.93) | 0.011 | 1.72 (1.33, 2.23) | 4E-05 | 0.74 (0.60, 0.90) | 0.004 |
|  | Comorbid | 848 | 2.57 (1.84, 3.58) | 3E-08 | 1.07 (0.83, 1.40) | 0.59 | 2.36 (1.80, 3.09) | 5E-10 | 0.84 (0.68, 1.04) | 0.12 |
| BioVU  (N = 46,072) | MDD-only | 5773 | 1.12 (1.09, 1.16) | 4E-13 | 1.06 (1.03, 1.10) | 1E-04 | 1.16 (1.12, 1.19) | < 1E-16 | 1.12 (1.09, 1.15) | 2E-16 |
|  | Anxiety-only | 4170 | 1.09 (1.05, 1.13) | 2E-06 | 1.06 (1.02, 1.10) | 0.003 | 1.12 (1.09, 1.16) | 2E-12 | 1.10 (1.07, 1.14) | 3E-09 |
|  | Comorbid | 4947 | 1.21 (1.17, 1.25) | < 1E-16 | 1.06 (1.03, 1.10) | 7E-04 | 1.25 (1.21, 1.28) | < 1E-16 | 1.16 (1.13, 1.20) | < 1E-16 |
| BioMe  (N = 9,076) | MDD-only | 1162 | 0.87 (0.53, 1.42) | 0.58 | 0.63 (0.39, 1.03) | 0.065 | 1.21 (0.84, 1.75) | 0.301 | 0.74 (0.51, 1.06) | 0.10 |
|  | Anxiety-only | 421 | 1.11 (0.81, 1.53) | 0.50 | 0.76 (0.56, 1.04) | 0.090 | 1.22 (0.94, 1.59) | 0.127 | 0.85 (0.66, 1.10) | 0.21 |
|  | Comorbid | 671 | 1.70 (1.14, 2.54) | 0.010 | 0.88 (0.59, 1.31) | 0.54 | 1.78 (1.30, 2.45) | 4E-04 | 0.80 (0.58, 1.09) | 0.16 |
| BioVU  (N = 11,734) | MDD-only | 1177 | 1.06 (0.99, 1.14) | 0.096 | 1.01 (0.94, 1.08) | 0.82 | 1.07 (1.00, 1.13) | 0.043 | 1.04 (0.98, 1.10) | 0.24 |
|  | Anxiety-only | 643 | 1.00 (0.91, 1.10) | 0.95 | 1.05 (0.96, 1.15) | 0.33 | 1.03 (0.95, 1.11) | 0.55 | 1.05 (0.97, 1.13) | 0.25 |
|  | Comorbid | 728 | 1.11 (1.01, 1.21) | 0.022 | 1.06 (0.97, 1.15) | 0.19 | 1.14 (1.05, 1.23) | 0.001 | 1.11 (1.03, 1.20) | 0.006 |

**Supplemental Table 6. PRS prediction of depression and anxiety case-only comparisons.** Results are show for PRSs for depression (MDD) and anxiety (ANX) predicting anxiety and depression comorbid groups compared to each other. Highlight indicates p-value < 0.01.

|  |  |  |  |  |  | **Models including both PRS** | | | | **Models including PRS at a time** | | | |
| --- | --- | --- | --- | --- | --- | --- | --- | --- | --- | --- | --- | --- | --- |
| **Site** | **Anc** | **Case group** | **N case** | **Control group** | **N Cont** | **MDD-PRS**  **OR (95% CI)** | **p-value** | **ANX-PRS**  **OR (95% CI)** | **p-value** | **MDD-PRS**  **OR (95% CI)** | **p-value** | **ANX-PRS**  **OR (95% CI)** | **p-value** |
| META | EUR | Comorbid | 14019 | MDD-only | 12201 | 1.09 (1.06, 1.12) | 7E-08 | 1.05 (1.02, 1.08) | 0.002 | 1.11 (1.08, 1.14) | 3E-13 | 1.09 (1.06, 1.12) | 5E-09 |
| META | EUR | Comorbid | 14019 | ANX-only | 10299 | 1.15 (1.11, 1.19) | 1E-16 | 1.02 (0.99, 1.05) | 0.21 | 1.16 (1.12, 1.19) | 1E-16 | 1.08 (1.05, 1.11) | 2E-07 |
| META | EUR | MDD-only | 12201 | ANX-only | 10299 | 1.06 (1.02, 1.09) | 0.001 | 0.97 (0.94, 1.01) | 0.11 | 1.04 (1.01, 1.07) | 0.006 | 1.00 (0.97, 1.02) | 0.74 |
| META | AFR | Comorbid | 1399 | MDD-only | 2339 | 1.07 (0.96, 1.19) | 0.24 | 1.05 (0.94, 1.17) | 0.36 | 1.09 (0.99, 1.20) | 0.074 | 1.06 (0.97, 1.17) | 0.21 |
| META | AFR | Comorbid | 1399 | ANX-only | 1064 | 1.12 (0.99, 1.26) | 0.078 | 1.03 (0.91, 1.16) | 0.64 | 1.12 (1.01, 1.25) | 0.037 | 1.06 (0.95, 1.18) | 0.27 |
| META | AFR | MDD-only | 2339 | ANX-only | 1064 | 1.05 (0.93, 1.17) | 0.44 | 0.98 (0.88, 1.09) | 0.70 | 1.03 (0.93, 1.13) | 0.59 | 1.00 (0.91, 1.10) | 0.98 |
| BioMe | AMR | Comorbid | 1343 | MDD-only | 1552 | 1.58 (0.99, 2.53) | 0.053 | 1.29 (0.87, 1.92) | 0.20 | 1.69 (1.17, 2.44) | 0.006 | 1.08 (0.79, 1.48) | 0.63 |
| BioMe | AMR | Comorbid | 1343 | ANX-only | 689 | 1.56 (1.07, 2.28) | 0.022 | 1.09 (0.79, 1.50) | 0.61 | 1.47 (1.07, 2.04) | 0.019 | 0.96 (0.73, 1.26) | 0.76 |
| BioMe | AMR | MDD-only | 1552 | ANX-only | 689 | 0.98 (0.62, 1.55) | 0.94 | 0.84 (0.57, 1.24) | 0.38 | 0.87 (0.61, 1.26) | 0.47 | 0.89 (0.65, 1.21) | 0.45 |
| MCB | EUR | Comorbid | 4929 | MDD-only | 4138 | 1.10 (1.05, 1.16) | 2E-04 | 1.11 (1.05, 1.17) | 9E-05 | 1.15 (1.10, 1.20) | 4E-09 | 1.15 (1.10, 1.21) | 1E-09 |
| MCB | EUR | Comorbid | 4929 | ANX-only | 2857 | 1.18 (1.12, 1.25) | 4E-09 | 1.03 (0.97, 1.09) | 0.30 | 1.20 (1.14, 1.26) | 3E-12 | 1.11 (1.05, 1.17) | 9E-05 |
| MCB | EUR | MDD-only | 4138 | ANX-only | 2857 | 1.07 (1.01, 1.14) | 0.014 | 0.93 (0.88, 0.99) | 0.014 | 1.04 (0.99, 1.10) | 0.12 | 0.96 (0.91, 1.01) | 0.12 |
| MGB | EUR | Comorbid | 3295 | MDD-only | 1297 | 1.07 (0.99, 1.15) | 0.95 | 1.05 (0.98, 1.14) | 0.17 | 1.09 (1.01, 1.17) | 0.018 | 1.08 (1.01, 1.16) | 0.03 |
| MGB | EUR | Comorbid | 3295 | ANX-only | 2249 | 1.15 (1.08, 1.23) | 2E-05 | 1.02 (0.95, 1.08) | 0.64 | 1.16 (1.09, 1.23) | 1E-06 | 1.07 (1.01, 1.14) | 0.024 |
| MGB | EUR | MDD-only | 1297 | ANX-only | 2249 | 1.08 (1.00, 1.17) | 0.055 | 0.96 (0.89, 1.04) | 0.36 | 1.07 (0.99, 1.15) | 0.089 | 0.99 (0.92, 1.07) | 0.83 |
| BioMe | EUR | Comorbid | 848 | MDD-only | 993 | 1.03 (0.66, 1.62) | 0.89 | 1.18 (0.83, 1.69) | 0.36 | 1.04 (0.73, 1.50) | 0.82 | 1.05 (0.79, 1.40) | 0.74 |
| BioMe | EUR | Comorbid | 848 | ANX-only | 1023 | 1.49 (0.95, 2.35) | 0.085 | 1.47 (1.03, 2.10) | 0.034 | 1.37 (0.94, 1.99) | 0.10 | 1.14 (0.85, 1.54) | 0.37 |
| BioMe | EUR | MDD-only | 993 | ANX-only | 1023 | 1.44 (0.93, 2.22) | 0.098 | 1.24 (0.89, 1.75) | 0.21 | 1.31 (0.92, 1.88) | 0.13 | 1.09 (0.82, 1.44) | 0.55 |
| BioMe | AFR | Comorbid | 671 | MDD-only | 1162 | 1.95 (1.04, 3.68) | 0.039 | 1.40 (0.74, 2.63) | 0.30 | 1.47 (0.90, 2.39) | 0.12 | 1.08 (0.67, 1.75) | 0.75 |
| BioMe | AFR | Comorbid | 671 | ANX-only | 421 | 1.53 (0.92, 2.54) | 0.105 | 1.16 (0.70, 1.92) | 0.57 | 1.46 (0.96, 2.20) | 0.074 | 0.94 (0.63, 1.41) | 0.77 |
| BioMe | AFR | MDD-only | 1162 | ANX-only | 421 | 0.78 (0.44, 1.40) | 0.41 | 0.83 (0.46, 1.48) | 0.52 | 0.99 (0.63, 1.55) | 0.97 | 0.87 (0.56, 1.36) | 0.54 |
| BioVU | EUR | Comorbid | 4947 | MDD-only | 5773 | 1.08 (1.03, 1.13) | 0.001 | 1.00 (0.95, 1.04) | 0.90 | 1.08 (1.04, 1.12) | 3E-04 | 1.03 (0.99, 1.08) | 0.11 |
| BioVU | EUR | Comorbid | 4947 | ANX-only | 4170 | 1.11 (1.06, 1.17) | 4E-05 | 1.00 (0.95, 1.05) | 0.91 | 1.11 (1.06, 1.16) | 2E-06 | 1.05 (1.01, 1.10) | 0.020 |
| BioVU | EUR | MDD-only | 5773 | ANX-only | 4170 | 1.03 (0.98, 1.08) | 0.26 | 1.01 (0.96, 1.06) | 0.81 | 1.03 (0.99, 1.08) | 0.16 | 1.02 (0.98, 1.06) | 0.39 |
| BioVU | AFR | Comorbid | 728 | MDD-only | 1177 | 1.04 (0.93, 1.17) | 0.46 | 1.05 (0.94, 1.17) | 0.38 | 1.07 (0.97, 1.18) | 0.19 | 1.07 (0.97, 1.18) | 0.16 |
| BioVU | AFR | Comorbid | 728 | ANX-only | 643 | 1.10 (0.97, 1.25) | 0.13 | 1.01 (0.89, 1.15) | 0.85 | 1.11 (0.99, 1.24) | 0.064 | 1.06 (0.95, 1.18) | 0.29 |
| BioVU | AFR | MDD-only | 1177 | ANX-only | 643 | 1.06 (0.94, 1.19) | 0.34 | 0.96 (0.86, 1.08) | 0.52 | 1.04 (0.94, 1.15) | 0.45 | 0.99 (0.90, 1.09) | 0.84 |

# **Supplementary References**

1. [Bielinski SJ, Chai HS, Pathak J, et al. Mayo Genome Consortia: a genotype-phenotype resource for genome-wide association studies with an application to the analysis of circulating bilirubin levels. *Mayo Clin Proc*. 2011;86(7):606-614.](http://paperpile.com/b/JX9KZN/nzmc)

2. [Das S, Forer L, Schönherr S, et al. Next-generation genotype imputation service and methods. *Nat Genet*. 2016;48(10):1284-1287.](http://paperpile.com/b/JX9KZN/uL5f)

3. [Belbin GM, Cullina S, Wenric S, et al. Toward a fine-scale population health monitoring system. *Cell*. 2021;184(8):2068-2083.e11.](http://paperpile.com/b/JX9KZN/RBDW)

4. [Howie BN, Donnelly P, Marchini J. A flexible and accurate genotype imputation method for the next generation of genome-wide association studies. *PLoS Genet*. 2009;5(6):e1000529.](http://paperpile.com/b/JX9KZN/pHcX)

5. [Dennis J, Sealock J, Levinson RT, et al. Genetic risk for major depressive disorder and loneliness in sex-specific associations with coronary artery disease. *Mol Psychiatry*. 2021;26(8):4254-4264.](http://paperpile.com/b/JX9KZN/X2Y2)

6. [Delaneau O, The 1000 Genomes Project Consortium, Marchini J. Integrating sequence and array data to create an improved 1000 Genomes Project haplotype reference panel. *Nature Communications*. 2014;5(1). doi:](http://paperpile.com/b/JX9KZN/Ie9z)[10.1038/ncomms4934](http://dx.doi.org/10.1038/ncomms4934)
